# Highly specific blockade of CCR5 inhibits leukocyte trafficking and reduces mucosal inflammation in murine colitis

**DOI:** 10.1038/srep30802

**Published:** 2016-08-05

**Authors:** Andrea Mencarelli, Sabrina Cipriani, Daniela Francisci, Luca Santucci, Franco Baldelli, Eleonora Distrutti, Stefano Fiorucci

**Affiliations:** 1 Singapore Immunology Network (SIgN), Agency for Science, Technology and Research (A*STAR), Singapore; 2 Dipartimento di Medicina, Università di Perugia, Piazza L. Severi 1, Perugia 06132, Italy; 3 Azienda Ospedaliera di Perugia, Perugia, Italy; 4 Dipartimento di Scienze Chirurgiche e Biomediche, Università di Perugia, Piazza L. Severi 1, Perugia 06132, Italy

## Abstract

Targeted disruption of leukocyte trafficking to the gut represents a promising approach for the treatment of inflammatory bowel diseases (IBDs). CCR5, the shared receptor for MIP1α and β and RANTES, is expressed by multiple leukocytes. Here, we aimed to determine the role of CCR5 in mediating leukocyte trafficking in models of colitis, and evaluate the therapeutic potential of maraviroc, an orally active CCR5 antagonist used in the treatment of CCR5-tropic HIV. Acute and chronic colitis were induced by administration of DSS or TNBS to wild-type and CCR5^−/−^ mice or adoptive transfer of splenic naïve CD4^+^ T-cells from wild type or CCR5^−/−^ mice into RAG-1^−/−^. CCR5 gene ablation reduced the mucosal recruitment and activation of CCR5-bearing CD4^+^ and CD11b^+^ leukocytes, resulting in profound attenuation of signs and symptoms of inflammation in the TNBS and transfer models of colitis. In the DSS/TNBS colitis and in the transfer model, maraviroc attenuated development of intestinal inflammation by selectively reducing the recruitment of CCR5 bearing leukocytes. In summary, CCR5 regulates recruitment of blood leukocytes into the colon indicating that targeting CCR5 may offer therapeutic options in IBDs.

Crohn’s disease and ulcerative colitis are two chronic inflammatory bowel diseases (IBDs) arising from inappropriate intestinal immune responsesto antigens derived from commensal microorganisms in genetically susceptible individuals. IBDs have traditionally been treated with non-specific immunosuppressants including corticosteroids and thiopurines, anti-inflammatory agents such as 5-aminosalicylic acid and more recently with tumor necrosis factor (TNF)α antagonists^1^. However, despite these treatments have been found effective in mild disease, a large proportion of patients with severe disease fail to achieve remission due to lack of drug response, loss of response, drug intolerance, or severe side effects that require cessation of therapy. There is therefore an urgent unmet clinical need for novel treatments that can suppress the intestinal inflammatory response in a more specific way, thereby overcoming the limitations of the currently available immunosuppressant therapies^1^.

A hallmark of chronic inflammatory disorders is the rapid recruitment and inappropriate retention of leukocytes at the site (s) of inflammation. Therapeutic targeting of the molecules that govern leukocyte recruitment and retention in the intestine is therefore a promising strategy for the treatment of IBDs^1^. Thus, the integrin α_4_β_7_ antagonist vedulizumab which attenuates inflammation by reducing leukocytes trafficking has gained approval for the treatment of IBD^1^. In addition to integrins, the recruitment of blood leukocytes to sites of inflammation is orchestrated by the interaction of chemokine receptors with specific ligands that are expressed in an anatomically restricted fashion^2^,^3^,^4^. More than 50 chemokines have been identified to date which are divided into four main families based on structural and functional characteristics^2^,^3^,^4^. The C-C chemokine receptor 5 (CCR5) is the shared receptor for the chemotactic mediators CCL3 (MIP-1α), CCL4 (MIP-1β) and CCL5 (RANTES). CCR5 is expressed by multiple cell lineages including T-cells, monocytes, macrophages and dendritic cells. Because expression of CCR5 ligands is increased in models of colitis, CCR5 antagonism might hold promise as a therapeutic strategy in IBDs^5^,^6^.

In addition to regulating leukocyte homing to the inflamed mucosa, CCR5 functions as a major co-receptor for HIV entry into target cells, and selective CCR5 antagonists have been developed that inhibit the replication of CCR5-tropic viral strains^7^,^8^. Maraviroc, is a small molecule that induces a non-competitive, slowly reversible, inhibition of CCR5 and displays therapeutic efficacy against CCR5-tropic HIV infection^9^,^10^,^11^. In addition, by blocking the signaling of all three CCR5 ligands, maraviroc effectively inhibits the migration and effector functions of CCR5-bearing leukocytes exerting anti-inflammatory and immune-modulatory effects^12^,^13^,^14^,^15^.

In the current report, we demonstrate that recruitment of CCR5-expressing leukocytes in the colon is essential for the onset and maintenance of inflammation in mouse models of colitis and that, maraviroc, a small molecule inhibitors of CCR5, protects against colitis development targeting CCR5-bearing leukocytes. These data suggest that CCR5 inhibitors could be used to modulate leukocyte trafficking in human IBDs.

## Materials and Methods

### Animals

CCR5^−/−^ (B6.129P2-Ccr5^tm1Kuz^/J), CCR5^+/+^, CX3CR1^+GFP^ on C57BL6 background and Rag-1^−/−^ mice were from the SPF animal facility at A*STAR, Singapore. The study was carried out in strict accordance with the guidelines of the Institutional Animal Care and Use Committee (IACUC) of the Biological Resource Centre (BRC) of Biopolis in Singapore or University of Perugia, Italy. The BRC IACUC protocol was approved by the National Advisory Committee for Laboratory Animal Research in Singapore (Permit Number: 110626), and by Perugia University’s ethical committee and Italian Ministry of Health permit no. 42/2014/B.

### DSS And TNBS Colitis Models

DSS colitis was induced by oral administration of 5% DSS in drinking water to weight matched, 4–6 weeks old male CD1 mice for 5 or 8 (not shown) days. Mice were randomized to receive DSS alone or DSS in combination with maraviroc at dose of 5, 25 or 50 mg/kg/day or prednisolone 5 mg/kg/day^16^, starting on day 1 after DSS. The dose of prednisolone used in this study has been demonstrated effective in reducing inflammation in rodent models of colitis and is roughly similar to the dose used in clinical setting (1 mg/kg)^16^. Animals were monitored daily for weight loss and fecal score (0, normal; 1, soft but still formed; 2, very soft; 3, diarrhea; and 4, liquid stool). The criteria/scoring system for assessing macroscopic damage was as follows; 0, no ulcer, no inflammation; 1, no ulcer, local hyperemia; 2, colon wall thickening/edema; 3, ulceration and inflammation at one site only; 4, two or more sites of ulceration and inflammation. Additional points were scored for the presence of indurations, intestinal adhesions, and each additional site of inflammation/ulceration >1 cm^2^ in surface area.

For TNBS colitis, weight matched, 4–6 weeks old male wild-type and CCR5^−/−^ mice were pretreatedwith 100 μl TNBS pre-sensitization solution (1% TNBS dissolved in 4:1acetone:olive oil solution) which was applied to shaved abdominal skin on day −7^17^. Control mice were subjected to the same protocol but using pre-sensitization solution that lacked TNBS. After 7 d, the mice were anesthetized by i.p. injection with ketamine/xylazine solution (100 mg/ml ketamine and 20 mg/ml xylazine in saline administered at 100 μl/10 g of body weight) and then treated with TNBS (0.5 mg per mouse) which was dissolved in 50% ethanol and administered intra-rectally using a 3.5 French catheter equipped with a 1 ml syringe. To ensure uniform distribution of TNBS throughout the colon and cecum, mice were held in a vertical position for 30 s after instillation. Mice were randomized into either the control group (TNBS only, n = 6) or treatment group (TNBS plus 50 mg/kg/d maraviroc per os, n = 5–6) from day 1 after TNBS administration, and then monitored daily as described above.

### T-cell transfer colitis model

Splenocytes were collected from 6–10 week old CCR5^−/−^ mice or wild-type controlmice (n = 8 per group) and naive CD4^+^ CD45RB^high^ T-cells were isolated by cell sorting. A total of 3 × 10^5 ^CD45RB^high^ cells were then injected intravenously into Rag1^−/−^ mice that were subsequently weighed and assessed for fecal score every 20 days to evaluate IBD development^18^. To investigate whether maraviroc rescues from intestinal inflammation induced by transfer colitis, Rag1^−/−^ mice were injected with CD4^+^ CD45RB^high^ T-cells and 34 days later randomized into either a control group (no further treatment, n = 6) or treatment with maraviroc, 50 mg/kg/d maraviroc per os (n = 4) for 3 weeks, 5 d/week.

### Flow cytometry and cell sorting

Cell suspensions were prepared from spleen, colonic lamina propria (LP) and mesenteric lymph nodes (MLN) and used for flow cytometry or cell sorting the next day. The following antibodies were obtained from Biolegend and used at 1:100 dilution unless specified otherwise; CD195 (CCR5)-APC or PE (clone HM-CCR5), CD44-PE/Cy7 (clone IM7), CD62L-FITC (clone MEL-14, BD Pharmingen), CD45-PE, FITC and PerCP-Cy5.5 (clone 30-F11 used at 1:200 dilution), CD4-APC/Cy7 or FITC or APC or AlexaFluor405 (clone RM4-5), CD45RB-APC/Cy7 (clone C36-16A), MHCII-APC/Cy7 (cloneM5/II4.152) GR1-Pacific blue (clone RB6-8C5, eBioscience) CD11b-PE/Cy7 and AlexaFlour700 (clone M1/70, eBioscience) Ly-6C-FITC (clone HK1.4), Ly-6G-PE (clone 1A8, BD Pharmingen) and DAPI. In the transfer colitis model, assessment of intracellular cytokine production by CD4^+^ T-cells was achieved by stimulating the sorted cells with PMA (0.1 μg/ml) and ionomycin (1 μg/ml) for 6.5 h *ex vivo* in the presence of Brefeldin A (10 μg/ml) for the final 5 h. The cells were then harvested and surface-labelled with CD4-APC/Cy7 before permeabilization using BD Cytofix/Cytoperm™ kits (BD Bioscience) and intracellular staining for 30 min at 4 °C using a 50 μl mixture of IL17-PE (clone TCII-18H101 used at 1:50 dilution), IFNγ-AlexaFluor^®^647 (clone XMG1.2 used at 1:50 dilution), TGF-β1-Pacific Blue (clone TW-16B4), and IL10-PE/Cy7 (clone JES5-16E3 used at 1:50 dilution).

### Inflammatory Histological Score

Sections of medial colon and small intestine were fixed in buffered formalin, routine 5 μm sections were prepared (~150 μm between each section, 4–8 per fragment), and then stained with hematoxylin and eosin. Stained sections were examined and scored in a blinded fashion. The histologic scoring system was as follows: A) *degree of inflammation* was graded semi-quantitatively from 0 to 4 (0, no signs of inflammation; 1, very low level; 2, low level of leukocyte infiltration; 3, high level of leukocyte infiltration, high vascular density, and thickening of the colon wall; and 4, transmural infiltration, loss of goblet cells, high vascular density, and thickening of the colon wall). B) *Extent of inflammation* from 0 to 4 (0 = none; 1 = mucosal; 2 = submucosal; 3 = mucosal + submucosal; 4 = full thickness). The total inflammatory score was obtained by multiplying the ‘degree’ by ‘extent’ scores (minimum 0, maximum score 16).

### ELISA assay

Lamina propria mononuclear cells (LPMC) were obtained from mice with TNBS-induced colitis (6 days duration) and seeded into 96 well plates (200,000 cells/well) for stimulation with LPS (5 μg/ml) in RPMI complete medium for 36 h at 37 °C^16^,^17^. Alternatively, CD4^+^ cells were sorted from the MLN after 3–4 d duration of TNBS colitis and then seeded into 96 well plates (100,000 cells/well) for stimulation with plate-bound anti-CD3 (5 μg/ml) and soluble anti-CD28 (2 μg/ml) in RPMI complete medium for 72 h at 37 °C. After incubation, the culture supernatant was analyzed for cytokine content by ELISA. Measurement of cytokine/chemokine release in the DSS model was achieved using a Bio-Plex^®^ Multiplex Immunoassay kit (BioRad, Italy).

### Statistical analysis

Data are expressed as mean ± standard error. Two-tailed, unpaired Student’s t tests were used to compare 2 groups of data, as indicated in the respective figures. P < 0.05 was considered significant. When more than two groups were considered, one way ANOVA followed by Bonferroni was used. GraphPad Prism software version 5.0 was used to prepare the graphics and perform all statistical analyses (GraphPad Software, San Diego, CA).

## Results

### Maraviroc Induces A Dose-Dependent Decrease In Mucosal Inflammation In DSS Colitis

**Figure 1:**
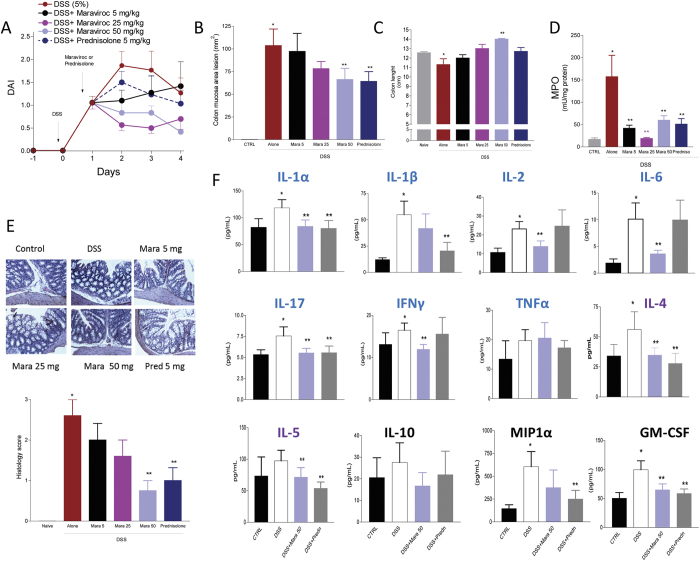
Maraviroc induces a dose-dependent decrease in mucosal inflammation in DSS Colitis. Mice were administered DSS (5%) in drinking water for 5 days and then treated with placebo or maraviroc (5, 25 or 50 mg/kg/d) or prednisolone (5 mg/Kg/d) for 4 days starting on day 2. **(A)** The disease activity index (DAI) was calculated daily for each individual mouse based on weight loss, rectal bleeding and stool consistency. **(B,C)** Macroscopic injury (lesion area in mm^2^) and colon length. **(D)** MPO activity (mU/mg protein). (**E)** Histopathology analysis of colon in mice administered DSS alone or in combination with maraviroc or prednisolone. Data Are mean ± SE of 5 mice per group. **(F)** Assessment of tissue biomarkers by Bio-Plex^®^ Multiplex immunoassay. Data were normalized to total tissue proteins. In each panel at least 5 animals per group are included. Markers for the Th1 phenotype are labeled in blue, for Th2 phenotype in violet, and for regulatory phenotype/chemokine in black. *P < 0.05 versus control; **P < 0.05 versus DSS.

In order to assess the efficacy of CCR5 blockade as a therapeutic approach in colonic inflammation, mice administered with DSS were treated with maraviroc (5, 25 or 50 mg/kg/day) or prednisolone (5 mg/kg/day) for the following 5 days. At a dose of 50 mg/kg/day, maraviroc attenuated the development of signs and symptoms of colitis with an efficacy that was comparable to 5 mg/kg/day prednisolone (Fig. 1). All three doses of maraviroc effectively blocked the recruitment of leukocytes into the colon, as assessed by measurement of MPO activity, although some of the therapeutic benefit of maraviroc treatment appeared to be lost at the lower doses (Fig. 1A–D). Maravirocalso protected against development of macroscopic and microscopic damage and colonic shortening, and was as effective as prednisolone at preventing DSS-induced changes in colonic levels of IL-2, IL-4, IL-5, IL-6, IL-17 and GM-CSF thought the some of these effects were likely of minor mechanistic relevance (Fig. 1E,F). These protective effect persisted for up to 8 days (data not shown).

### CCR5 Gene Ablation Or Maraviroc Therapy Reduces Inflammation In TNBS Colitis

**Figure 2:**
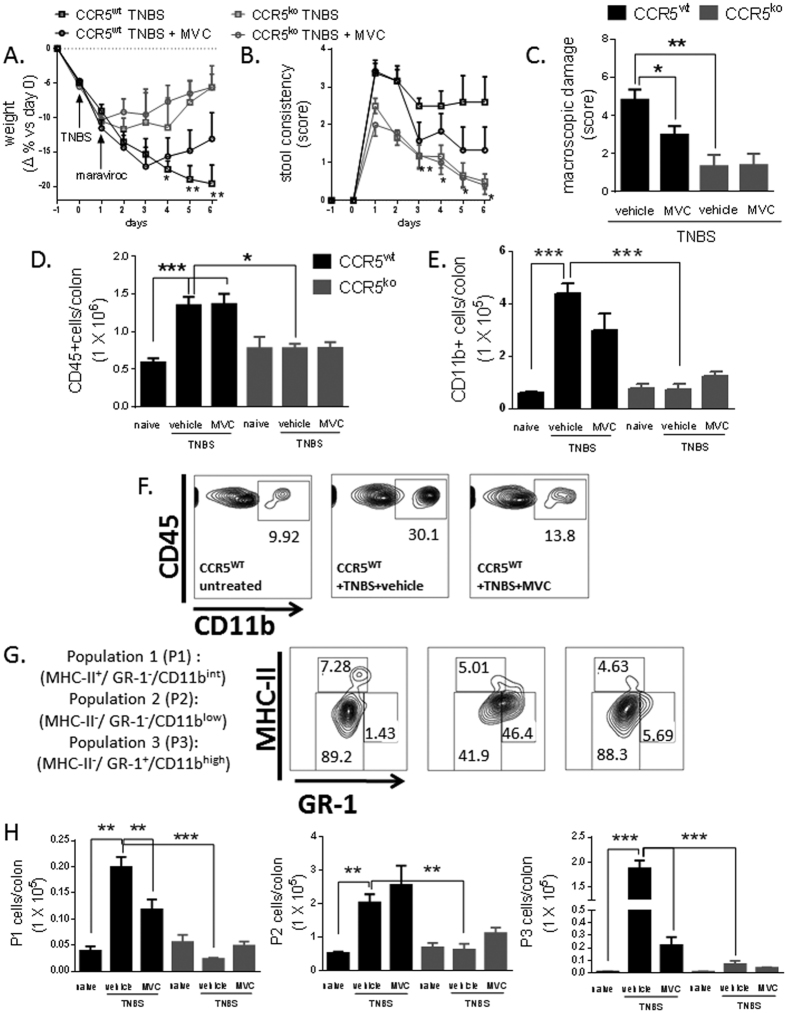
CCR5-knockout mice are protected against TNBS-induced acute colitis. (**A,B**) Severity of TNBS-induced colitis (weight loss and stool consistency) was reduced in CCR5^−/−^ mice. (**C**) Macroscopic inflammation. n = 6 per group (*P < 0.05, **P < 0.01, ***P < 0.005). (**D**) Absolute number of CD45^+^ and CD11b^+^ cells detected in the colonic LP of individual naïve and coltic mice. (**E**) Representative flow cytometry analysis of CCR5^+/+^ mice treated with TNBS showing staining of total colonic leucocytes for CD11b+ cells (upper panel, after exclusion of MHC-II^high^ CD11c^high^ putative dendritic cells) and their relative expressions of MHC-II and GR1 (lower panel). **(F**) Absolute number of cells in the colonic LP corresponding to Population 1 (P1) (MHC-II^+^/Gr-1^−^/CD11b^int^), Population 2 (P2) (MHC-II^−^/Gr-1^−^/CD11b^low^) and Population 3 (P3) (MHC-II^−^/Gr-1^+^/CD11b^high^) in untreated and TNBS coliticmice that were treated or not by oral administration of maraviroc. Data shown mean ± SE n = 3 per group (*P < 0.05, **P < 0.01, ***P < 0.005; two-tailed, unpaired Student’s t test).

We next investigated the involvement of CCR5 in the pathology of a T-cell-driven colon inflammation using a TNBS-induced model of colitis in both wild-type mice and CCR5^−/−^ animals that received either placebo or maraviroc treatment (50 mg/kg for 5 days, starting one day after colitis induction) (Fig. 2A–C). In wild-type animals, TNBS-induced colitis was associated with a robust influx of CD45^+^ CD11b^+^ leukocytes into the LP (Fig. 2D,E). Analysis of the expression of MHC-II and myeloid differentiation antigen Gr-1 in these cells allowed the identification of three major subsets (Fig. 2F): population 1 comprised MHC-II^+^ Gr-1^−^ CD11b^int^ cells (P1; putative macrophages), whereas population 2 was made-up of MHC-II^−^Gr-1^−^CD11b^low^ cells (P2; putative monocyticcells), and population 3 cells were MHC-II^−^Gr-1^+^ CD11b^high^ (P3; putative granulocytes). Further analysis of Ly6C, Ly6G, CD11c and CX3CR1 expression among the LP infiltrating leukocytes indicated that population P2 included a substantial fraction of Ly6C+ i.e. blood derived monocytes, and that exposure to TNBS increased substantially the proportion of these cells (Table 1 and Supplementary Figure 1)^19^,^20^. In wild-type animals, leukocyte infiltration of the colon was associated with development of phenotypic features of acute colitis (Fig. 2A–C). In contrast, CCR5^−/−^ mice exhibited milder weight-loss, reduced severity of diarrhea, and only limited thickening of the colon wall compared with CCR5^+/+^ animals (Fig. 2A–C; P < 0.05 versus wild type). Importantly, maraviroc rescued wild-type animals from the development of wasting disease, diarrhea and macroscopic inflammation (Fig. 2A–C; P < 0.05 versus DSS wild type). Consistent with the concept that CCR5 blockade achieves these beneficial effects by impairing the mucosal recruitment of multiple leukocyte lineages, we also observed that disruption of CCR5 signaling either by gene ablation or small molecule antagonism significantly attenuated the influx of myeloid cells into the LP (Fig. 2D–F).

### CCR5 Expression In Both The Innate And Adaptive Immune Compartments Contributes To Murine Intestinal Inflammation

**Figure 3:**
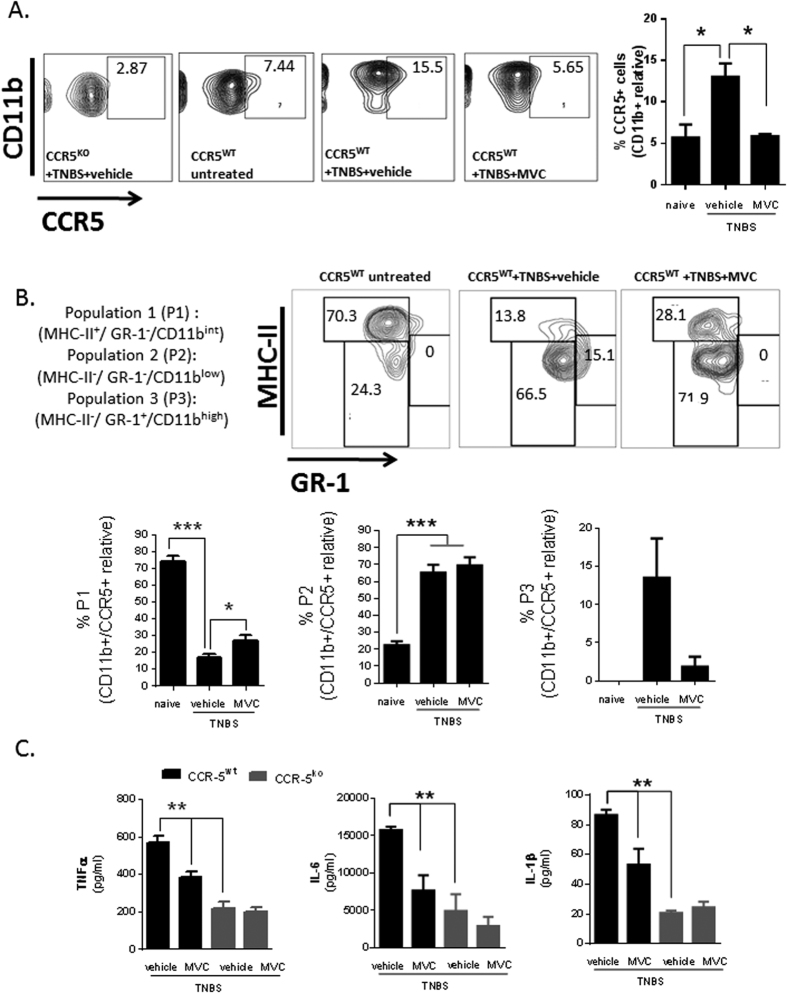
CCR5 expression in myeloid cells in steady state condition and during colitis. **(A**) CD11b^+^ cells in the colonic LP exhibit up-regulation of CCR5 during colitis which can be blocked by administration of maraviroc therapy (n = 3 per group; *P < 0.05). (**B**) The CD11b^+^ CCR5^+^ cell colonic infiltrate was analyzed for differential expression of MHCII and GR1; the upper panel shows a representative dot plot and the lower panel shows the relative proportions of P1, P2 and P3 cells detected in untreated and colitic mice that were treated or not with maraviroc. (**C**) Cytokine production by colonic LP leukocytes upon *ex vivo* stimulation with LPS for 36 h. Values indicate mean ± SE; n = 3/group (*P < 0.05, **P < 0.01, ***P < 0.005).

Further analysis of the CD11b^+^ myeloid cells detected in wild-type colitic mice revealed an increase in CCR5 expression among these cells after exposure to TNBS (Fig. 3A). This increase in CCR5 expression in the myeloid cell compartment was accompanied by a shift away from the predominant macrophage population detected in the steady-state (CCR5^+^ CD11b^int^ MHC-II^+^ Gr-1^−^ cells), towards an inflammatory infiltrate made up primarily of monocyte-like cells (CCR5^+^ CD11b^low^ MHC-II^−^Gr-1^−^) (Fig. 3B; *p < 0.05, ***p < 0.005). CCR5 antagonism using maraviroc therapy abrogated the accumulation of CCR5^+^ CD11b^+^ leukocytes in the inflamed colonic mucosa (Fig. 3A and Supplementary Figure 2, *p < 0.05), and was associated with a robust reduction of pro-inflammatory mediators including TNFα, IL-6 and IL-1β (Fig. 3C).

**Figure 4:**
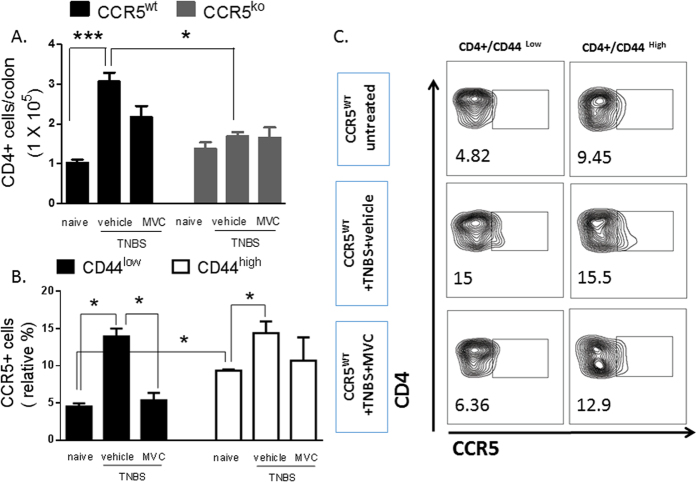
CCR5 expression by colonic CD4+ T-cell subsets is up-regulated in colitis and decreased by maraviroc therapy. **(A**) Absolute number of CD4^+^ T-cells detected in the colonic LP of mice after 6 days of TNBS colitis with or without maraviroc therapy for the final 5 days. **(B**) CCR5 expression level within the CD44^low^ and CD44^high^ populations of colonic CD4^+^ T-cells. (**C**) Representative contour plots showing CD44 and CCR5 expression levels among CD4^+^ T-cells in the colonic LP in both control and colitic mice that were treated or not with maraviroc or vehicle. Values are mean ± SE; n = 3 (*P < 0.05).

Importantly, CCR5 also contributed to the mucosal recruitment of pro-inflammatory T cells. After 6 days of TNBS administration, we detected a substantial number of CD4^+^ cells in the LP, but only in mice that were sufficient in CCR5 (Fig. 4A). CD44 expression has previously been described as a marker of memory T-cells with tissue homing potential^21^.

CCR5 is considered to be a phenotypic marker for effector/memory T-cells^22^,^23^. Consistent with this view, CD4+ T-cells with a CD44^high^ ‘memory’ phenotype exhibited stable high expression of CCR5 in the mouse LP (Fig. 4B). Furthermore, TNBS colitis associated with increased levels of CCR5 expression within the T-cell pool, but this was largely restricted to the CD44^low^ immature subset, suggesting maturation of these cells towards an effector/memory phenotype during inflammation (Fig. 4B). CCR5 upregulation by colitis in CD4+ CD44^low^ cells was abrogated by co treatment with maraviroc (Fig. 4B,C).

### Mucosal Inflammatory Responses Of CCR5^+^ Leukocytes Exhibit An Effector/Memory Th17 Profile

**Figure 5:**
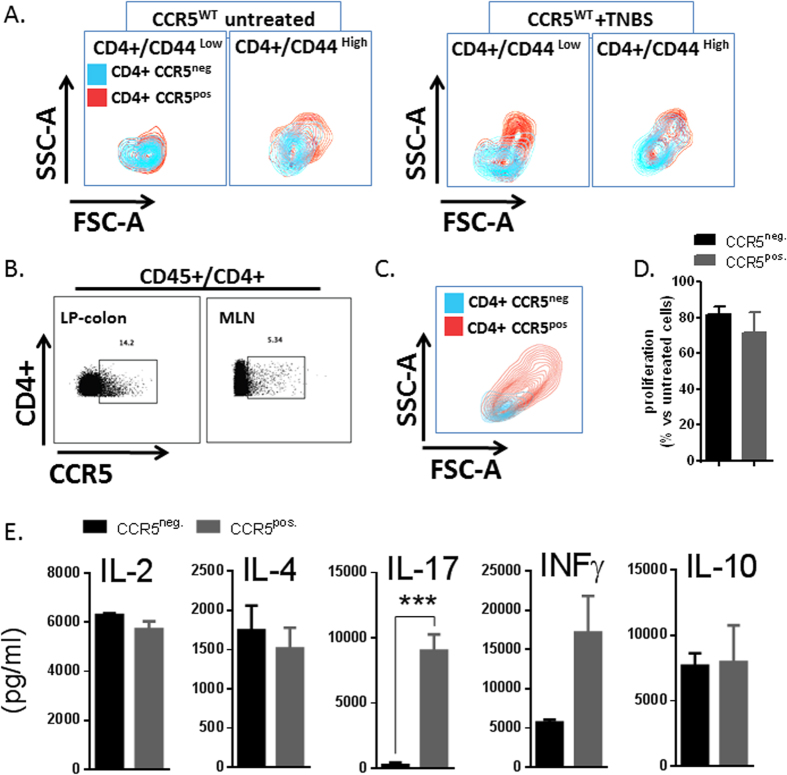
Differential morphology and CCR5 expression by memory CD4^+^ T-cells during TNBS colitis. (**A**) CD4+ T-cell morphology of the CCR5+ subset (red contour) and CCR5- subset (blue contour) within the CD44^high^ and CD44^low^ populations of colonic CD4+ T-cells obtained from mice that were either untreated or subjected to TNBS colitis for 7 days. (n = 3–4/group; *P < 0.05, **P < 0.05). (**B**) Contour plot showing the percentage of CCR5^+^ cells with the CD4^+^ T-cell pool of colonic LP and mesenteric lymph nodes after 3–4 days of TNBS colitis. (**C)** CD4+ T-cell morphology of the CCR5+ subset (red contour) and CCR5- population (blue contour) obtained from mesenteric lymph nodes of TNBS colitic mice. Proliferation (**D)** and cytokine production (**E**) by CCR5+ and CCR5- populations of sorted CFSE-labeled CD4+ T-cells after *in vitro* stimulation with CD3/CD28 for 72 hours. Values are mean ± SE; n = 4 (***P < 0.005).

Within the CD44^high^ subset of LP CD4^+^ T-cells, we further identified that CCR5^+^ cells were substantially larger in size than CCR5^−^ cells in both the steady-state and during inflammation, suggesting selective cell cycle entry and clonal expansion of the CCR5^+^ population in this model^24^. Similarly, while the CD44^low^ subset of CD4^+^ LP exhibited fairly uniform size in the steady-state, there was a robust increase in the size of the CCR5^+^ cells following exposure to TNBS (Fig. 5A). These data are consistent with the known ability of chemokines to influence T-cell differentiation^25^.

Previous reports suggest that CCR5 is selectively expressed on IFN-γ producing Th1 cells and allowing targeted recruitment of these cells to inflammatory sites^23^,^24^. Therefore we next sought to determine whether CCR5-expressing leukocytes in the TNBS model produce cytokines consistent with a Th1 phenotype. For this purpose, we FACS-sorted CCR5- and CCR5+ populations of CD4+ T-cells from the LP and MLN of colitic mice and then stimulated these cells with anti-CD3/CD28 mAb. Of relevance, we found that almost all colonic CCR5^+^ CD4^+^ T-cells died during the 3-day stimulation period (data not shown), likely due to activation-induced cell death, whereas there was no difference in the rate of cell death and proliferation detected among MLN-derived cells (data not shown and Fig. 5D, respectively). Assessment of cytokine levels in culture supernatants of MLN-derived cells demonstrated that CCR5^+^ CD4^+^ cells displayed a profile consistent with Th17 polarization during TNBS colitis (Fig. 5E).

### Leukocyte Expression Of CCR5 Is Required For Both Acute And Chronic Murine Colitis

**Figure 6:**
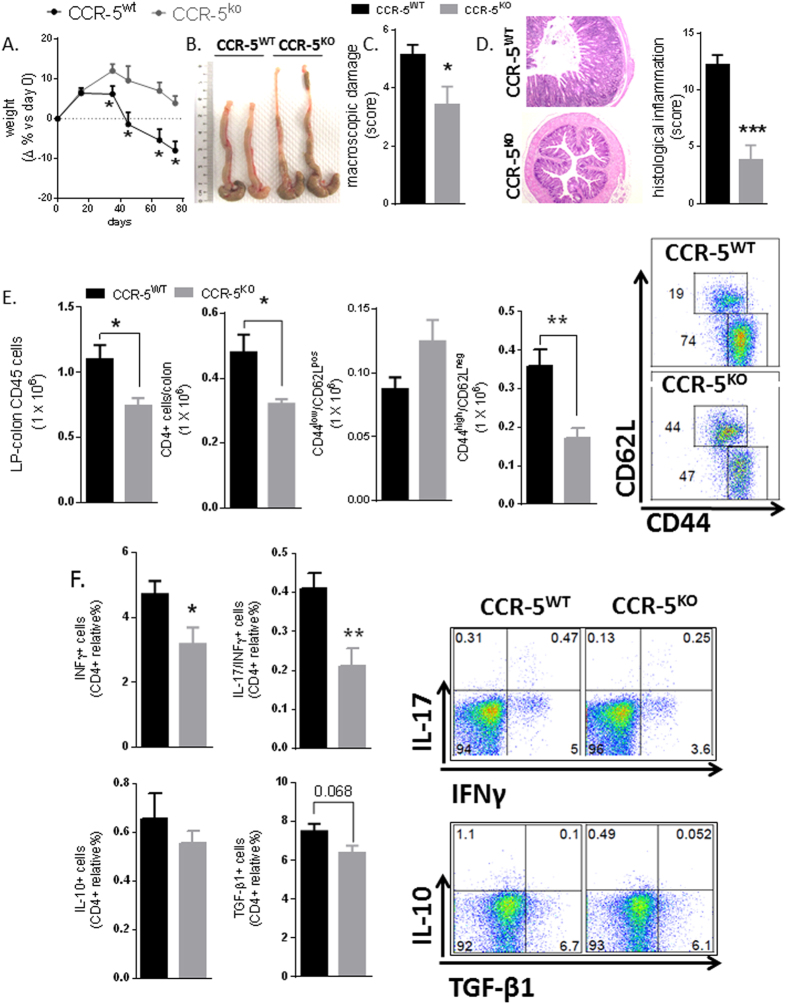
CCR5^+^ CD4^+^ T-cells are critically required for the onset and maintenance of chronic colitis. **(A–D**) Colitis severity (weight loss, stool consistency, macroscopic and microscopic inflammation and colon length) and representative photo of mouse colons after adoptive transfer of CD4^+^ T-cells from the spleens of CCR5^+/+^ and CCR5^KO^ mice into Rag1^−/−^ recipient animals (3 × 10^5^ CD45RB^high^ cells/mouse). Values indicate mean ± SE; n = 7 (*P < 0.05). (**E**) Phenotypic analysis of colonic leukocytes showing the total number of CD45+ cells, total CD4+, CD4+ naïve cells (CD62L^pos^/CD44^low^), and CD4+ activated/memory cells (CD62L^neg^/CD44^high^), with representative dot plots showing expression of CD62L and CD44 within the CD4+ T-cell compartment. (**F**) Cytokine production by CD4^+^ T-cells obtained from LP of colitic mice. Values are mean ± SE; n = 6/group (*P < 0.05, **P < 0.01).

The role of CCR5 in the context of chronic inflammation was investigated using the adoptive transfer model of T-cell-mediated colitis in animals grafted with either CCR5^+/+^ or CCR5^−/−^ T-cells. Adoptive transfer of naïve CD4^+^ CD45RB^high^ T-cells from wild-type miceinto RAG1^−/−^ recipients conferred gut pathology characterized bya severe weight loss, diarrhea, macroscopic inflammation and colonic thickening (Fig. 6A–D). In contrast, mice that were transferred with naïve CD4^+^ CD45RB^high^ T-cells that lacked CCR5 exhibited milder disease and fewer total colonic leukocytes as well as reduced infiltration of CD4^+^ effector/memory T-cells into the gut mucosa (Fig. 6E). Indeed, unlike CD4^+^ T-cells from wild-type donors, CCR5^−/−^ CD4^+^ T-cells appeared unable to efficiently mature into effector cells and exhibited significantly reduced production of IFNγ and IL-17 when analyzed *ex vivo* (Fig. 6F). A similar immune profile was also observed in the MLN of mice transferred with CCR5^−/−^ CD4^+^ T-cells (Supplementary Figures 3 and 4). Together, these data indicated that CCR5 ablation in CD4^+^ T-cells abolishes a Th1/Th17-type inflammatory pathology of the gut which resembles that of human IBD^26^.

### CCR5 blockade attenuates sign and symptoms of chronic colitis

**Figure 7:**
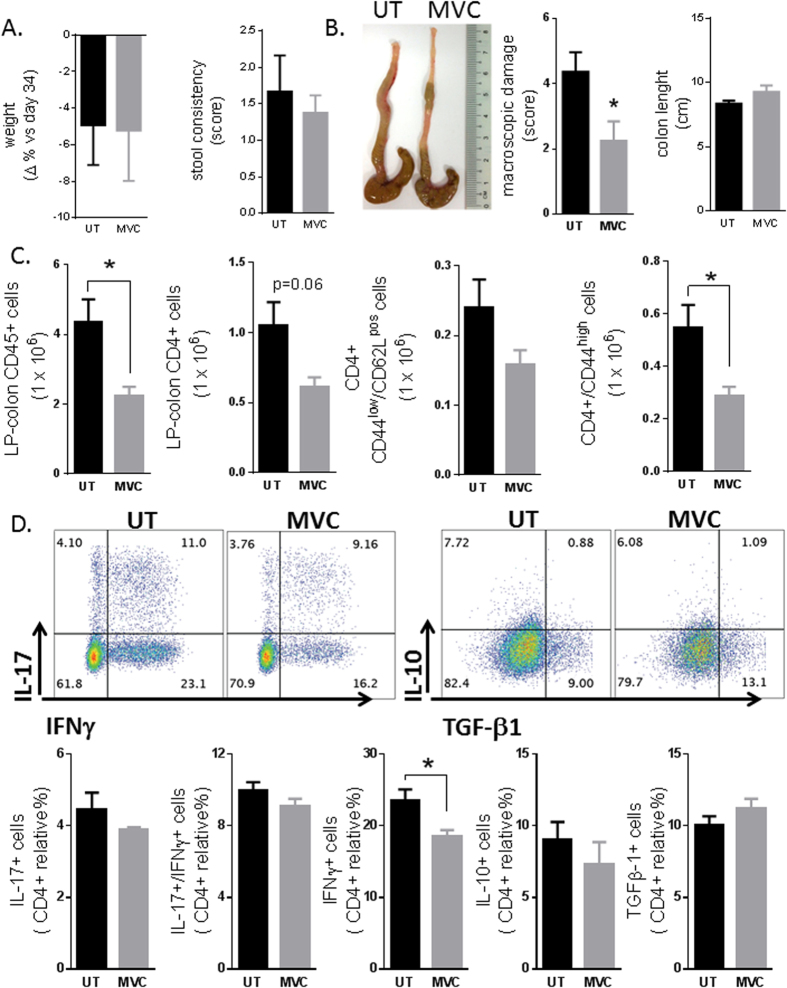
CCR5 Inhibition reduces chronic colitis severity. **(A,B**) Colitis severity (weight loss from the day of maraviroc treatment, stool consistency, macroscopic inflammation and colon length) and representative photo of mouse colons after adoptive transfer of CD4^+^ T-cells from the spleens of CCR5^+/+^ mice into Rag1^−/−^ recipient animals (3 × 10^5^ CD45RB^high^ cells/mouse) alone or after 3 weeks of treatment with maraviroc (50 mg mg/kg/d, 5 days for week). Values indicate mean ± standard error of n = 4 and 6 per group (*P < 0.05). (**C**) Phenotypic analysis of colonic leukocytes showing the total number of CD45+ cells, total CD4+, CD4+ naïve cells (CD62L^pos^/CD44^low^), and CD4+ activated/memory cells (CD44^high^). (**D**) Cytokine production by CD4^+^ T-cells obtained from LP of colitic mice alone or treated with maraviroc. Values indicate mean ± standard error of n = 4/group (*P < 0.05).

To investigate whether the therapeutic effects observed in acute models of colitis was maintained over time, Rag1^−/−^ rendered colitic by transfer of CD4^+^ T cells from wild type donors, were administered with maraviroc 50 mg/kg/d for 3 weeks starting on day 34 after colitis induction. As shown in Fig. 7 and Supplementary Figure 5, we found that maraviroc attenuates colon inflammation in this setting and reduced signs and symptoms of colitis, as well as the inflow of CD45+ cells into the LP. Analysis expression of markers of activation demonstrated that long term administration of maraviroc reduced the number of IFNγ+ cells, but had no effect on IL-10 and TGFβ stained cells. Altogether these data confirm that the maraviroc attenuates inflammation in chronic models of colitis.

## Discussion

In the present study we provide evidence that CCR5, a chemokine receptor expressed by different leukocytes exerts a critical role in the development of acute and chronic inflammation in murine models of colitis. Analysis of CCR5 expression on LP leukocytes in mice administered TNBS revealed that colitis development in this modelis mediated by the recruitment of three major leukocyte populations: MHC-II^+^ Gr-1^−^ CD11b^int^ cells (putative macrophages), MHC-II^−^Gr-1^−^ CD11b^low^ cells (likely monocytes/macrophages), and MHC-II^−^ Gr-1^+^ CD11b^high^ cells (putative granulocytes). A further characterization of these cells, revealed that the monocytes/macrophages compartment in the TNBS model is largely composed by Ly6C+ cells. Previous studies have shown that in the steady state the intestinal monocytes/macrophages population in mice is maintained by the continuous recruitment of Ly6C+ monocytes (CD14+ cells in humans) from the blood stream^25^. Under resting conditions, these monocytes undergo local differentiation into reparative, anti-inflammatory, macrophages (MHCII+ CD11b^Int^ and CX3CR1^+^) that are highly phagocytic, hypo-responsive to pro-inflammatory stimuli producing large amounts of IL-10 in inflammatory milieus. The maturation of these Ly6C^high^ blood monocytes into tissue-resident macrophages (MHCII^pos^ Gr1^neg^ CD11b^+^) is governed by signals received from the local micro environment, and involves cell transition through several intermediate phenotypes. However, the normal pattern of monocyte differentiation is disrupted during inflammation, leading to the recruitment and accumulation of Ly6C^+^ MHCII^pos^ CX3CR1^int^ effector monocytes in the intestine, where theycan generate TNFα and other soluble mediators. In the present study, we observed that in the steady state themonocytic cell population of the colonic LP is largely made up byLy6C^+^ cells (up to 60%), and the predominance of Ly6C+ cells in this compartment increases further in the TNBS colitis (>90% of the monocytic cell infiltrate), strongly indicating the selective recruitment of Ly6C+ effector monocytes from the blood stream^23^,^24^. Consistent with this view, while only 10% of gut monocytic cells expressed CCR5 in the steady state, the proportion of CCR5+ cells increased to ~70% in response to TNBS, supporting the notion that colitis development in this model is supported by recruitment of circulating Ly6C^+^ monocytes via a mechanism that involves CCR5. Importantly, these Ly6C^+^ CCR5^+^ cells displayed a strong polarization towards an effector phenotype and produced pro-inflammatory cytokines including IL-1β, IL-6 and TNFα.

In addition to regulating the trafficking of innate leukocytes, CCR5 is also critically required for the recruitment of circulating T-cells to the intestinal mucosa. Thus CCR5^−/−^ mice were essentially unable to recruit CD4^+^ cells into the LP in response to TNBS^27^. Phenotypic and functional characterization of LP lymphocytes demonstrated that ~15% of CD4^+^ cells in the gut express CCR5 in the steady state, and that exposure to TNBS promoted the recruitment of additional CCR5+ CD4+ T-cells with robust polarization towards a Th17 profile. These data underlined a broad role of CCR5 in regulating the mucosal recruitment and functional maturation of both innate immune cells and T-cells arriving from the blood.

Consistent with the data obtained from chemical colitis models, the adoptive transfer of naive CD4^+^ CD45RB^high^ T-cells into RAG-1^−/−^ mice demonstrated that CCR5-deficent T-cells were unable to replicate the chronic colitis that was induced by CCR5-sufficient T-cells. Transplantation of CCR5^−/−^ T-cells associated with a substantial reduction in the number of CD4^+^ and CD45^+^ cells recruited in the LP of grafted animals, as well as reduced weight loss, lower diarrhea score, milder macroscopic inflammation and reduced colonic shortening. In contrast to the acute chemical colitis models, attenuation of gut inflammation by CCR5 gene ablation, however, was due largely to the impaired infiltration of CD4^+^ T-cells and did not depend on changes in the CD11b^+^ myeloid compartment. In addition to disrupting the mucosal recruitment of CD4^+^ T-cells, genetic ablation of CCR5 also reducedthe proportion of cells with an activated phenotype (CD44^high^CD62L^−^), and impaired the ability of these cells toproduce IFNγ and IL-17 without any discernable loss of immuno regulatory function (IL-10+ TGF-β+ CD4+ T-cell frequency).

The critical role of CCR5 in regulating leukocyte trafficking in intestinal inflammation was confirmed by the protective effects exerted by maraviroc in three model of colitis, i.e. the acute colitis induced by DSS and TNBS and in the chronic colitis induced by transfer of CD4+ T cells into Rag1^−/−^ mice. Maraviroc is an orally active, allosteric modulator of CCR5 that binds to the extracellular portion of the receptor, thereby preventing the conformational changes required for signal transduction after engagement of physiological ligands (CCL3, 4 and 5) or viral proteins (HIV GP120)^8^,^9^. Here, we have shown that administration of maraviroc attenuates gutinflammation in rodent models of colitis by selectively depleting CD11b^+^ CCR5^+^ cells from the colonic mucosa (in the TNBS model) and modulating the production of signature cytokines such as IL -1β, IL-6 and TNFα in both DSS-induced and TNBS models of colitis. Of relevance, the therapeutic effect of maraviroc was maintained in the chronic model of colitis resulting in a robust attenuation of recruitment of CD45+/CD11b+ cells in the LP.

While the data we report strongly support a role for CCR5 in rodent colitis, CCR5 gene mutations do not overtly modulate human IBD phenotypes, as demonstrated by the fact thata common 32-bp ‘loss-of-function’ deletion (Δ32) of CCR5 fails to confer protection against IBD^28^,^29^,^30^,^31^,^32^,^33^. In contrast, the Δ32 mutation confers protection against development of primary sclerosing cholangitis in IBD^34^. These data suggest that alternative mechanisms of leukocyte trafficking to the gut may compensate for the absence of CCR5 signaling in IBD patients with this mutation, and conversely that the benefits of maraviroc therapy may be restricted to IBD patients with a functional CCR5 gene.

In conclusion, we have provided compelling evidence that CCR5 mediates the trafficking of both innate and adaptive immune cells in rodent models of colitis. Pharmacological inhibition or genetic ablation of CCR5 rescued mice from colitis in both acute and chronic models, hence the clinically-approved small molecule antagonist of CCR5, maraviroc may represent a novel approach to reducing the mucosal trafficking of blood leukocytes in human IBDs.

## Additional Information

**How to cite this article**: Mencarelli, A. *et al*. Highly specific blockade of CCR5 inhibits leukocyte trafficking and reduces mucosal inflammation in murine colitis. *Sci. Rep.* **6**, 30802; doi: 10.1038/srep30802 (2016).

## Supplementary Material

Supplementary Information

## Figures and Tables

**Table 1:** Myeloid markers expression in Population 1, 2 and 3.

Markers	Naïve mice			TNBS Colitis		
	Population 1 (%)	Population 2 (%)	Population 3 (%)	Population 1 (%)	Population 2 (%)	Population 3 (%)
Ly6G+	ND	ND	ND	ND	ND	100
Ly6C+	6.6 ± 2	60.5 ± 4	ND	*26.8 ± 5	*87.2 ± 2	100
Ly6C^+^ CD11c^low^	3.9 ± 2	2.46 ± 1	ND	*18.2 ± 2	1.7 ± 1	ND
CD11c^low^	29.1 ± 1	10.1 ± 1	ND	23.1 ± 5	*2.84 ± 1	ND
CX3CR1+	42.7 ± 2	23.5 ± 1	ND	22 ± 5.6	*6.4 ± 0.5	2.6 ± 0.7

Flow-cytometric analysis of the myeloid cell markers Ly6-G, Ly6-C, CD11c and CX3CR within population P1 cells (MHC-II^+^ GR-1^+^ CD11b^int^ ), P2 cells (MHC-II^−^GR-1^−^CD11b^low^) and P3 cells (MHC-II^−^GR-1^+^ CD11b^high^). CX3CR1 expression was determined by analysis of the colonic leukocyte infiltrate in CX3CR1^+GFP^ mice. Values indicate mean ± standard error of n = 3 per group (*P < 0.05, two-tailed, unpaired Student’s t test).

% of positive cells; ND, not detected.
