# Supplementary material for: Highly specific blockade of CCR5 inhibits leukocyte trafficking and reduces mucosal inflammation in murine colitis
Source: Sci Rep. 2016 Aug 5;6:30802. doi: 10.1038/srep30802 (PMC4974621; doi:10.1038/srep30802)
Supplement: Supplementary Information [file srep30802-s1.pdf]

Highly specific blockade of CCR5inhibits leukocyte trafficking and reduces mucosal inflammation in murine colitis

Mencarelli, Andrea PhD[1], Cipriani, Sabrina PhD[2], Francisci, Daniela MD[2], Santucci, Luca MD[3]
Baldelli, Franco MD[2], Distrutti, Eleonora MD[3], Fiorucci, Stefano, MD[4]

Legend to Supplementary Figures

Supplementary Figure 1. *Flow cytometric analysis of CD11b+ cells in colonic LP of untreated and TNBS colitic mice.*

A) Percentage of $CD11b^{+}$ cells within the CD45+ colonic leukocyte pool in untreated wild-type mice and $CCR5^{-/-}$ mice that were subjected to TNBS colitis (or vehicle control) and treated with maraviroc (or vehicle control). The proportions of Population 1 cells ($MHC\text{-}II^{+}$/ $GR\text{-}1^{-}$/$CD11b^{int}$), Population 2 cells ($MHC\text{-}II^{-}$/ $GR\text{-}1^{-}$/$CD11b^{low}$) and Population 3 cells ($MHC\text{-}II^{-}$/ $GR\text{-}1^{+}$/$CD11b^{high}$) is indicated. **B**) Relative cellular size of the P1, P2 and P3 populations (red dot plots) as determined in $CCR5^{+/+}$ TNBS-treated mice. Values indicate mean ± standard error of n=3 per group (*P<0.05, **P<0.01,***P<0.005; two-tailed, unpaired Student's t test).

Supplementary Figure 2. *CCR5 expression in colonic myeloid cells.*

A) Absolute number of $CCR5^{+}$ cells belonging to the CD11b+ myeloid pool, Population 1, Population 2 or Population 3. Percentage of CCR5, isolated from each LP-colon obtained from untreated and colitic mice treated with vehicle or maraviroc (MARAVIROC). Values indicate mean ± standard error of n=3 per group (*P<0.05, **P<0.01,***P<0.005; two-tailed, unpaired Student's t test). **B**) Representative contour plot showing CD44 and CCR5 expression by CD4+ T-cells cells obtained from colonic LP of $CCR5^{-/-}$ mice that were either subjected to TNBS colitis or left untreated.

Supplementary Figure 3. *CD4+ T-cell analysis during adoptive transfer colitis.*

Percentage of total $CD4^{+}$ T-cells, $CD4^{+}$ naïve T-cells ($CD62L^{pos}CD44^{low}$) and $CD4^{+}$ activated/memory T-cells ($CD62L^{neg}$/$CD44^{high}$) detected in the colonic LP (**A**) and MLN (**B**) of recipient animals after engraftment with $CD4^{+}$ naive T cells sorted from the spleens of $CCR5^{+/+}$ and $CCR5^{KO}$. Values indicate mean ± standard error of n=6 per group (**P<0.01, two-tailed, unpaired Student's t test).

Supplementary Figure 4. *Immunophenotype of MLN cells in the adoptive transfer colitis model.*

A) Representative photo of MLN and numbers of $CD45^{+}$ leukocytes and $CD4^{+}$ T-cells detected upon completion of the adoptive transfer colitis protocol. Values indicate mean ± standard error of n=7 per group (*P<0.05, two-tailed, unpaired Student's t test. **B**) Dot plots showing staining of MLN $CD4^{+}$ T-cells with antibodies against CD62L and CD44, as well as the absolute numbers of CD4+

naïve T-cells ($CD62L^{pos}CD44^{low}$), and CD4+ activated/memory T-cells ($CD62L^{neg}CD44^{high}$) detected. **C**) Cytokine production by $CD4^+$ T-cells obtained from MLN of colitic mice. Values indicate mean ± standard error of n=6 /group (*P<0.05, **P<0.01, two-tailed, unpaired Student's t test).

Supplementary Figure 5. *Immunophenotype of LP-colon and MLN cells in the adoptive transfer colitis model, during maraviroc treatment.*

A) Percentage of total $CD4^+$ T-cells, $CD4^+$ naïve T-cells ($CD62L^{pos}CD44^{low}$) and $CD4^+$ activated/memory T-cells ($CD44^{high}$) detected in the colonic LP. (**B**) MLN numbers of $CD45^+$ leukocytes and $CD4^+$ T-cells and percentage of $CD4^+$ T-cells, $CD4^+$ naïve T-cells ($CD62L^{pos}CD44^{low}$) and $CD4^+$ activated/memory T-cells ($CD44^{high}$) detected upon completion of the adoptive transfer colitis protocol, in combination with the administration of maraviroc for 3 weeks. Values indicate mean ± standard error of n=4 and 6 per group (*P<0.05 and**P<0.01, two-tailed, unpaired Student's t test).

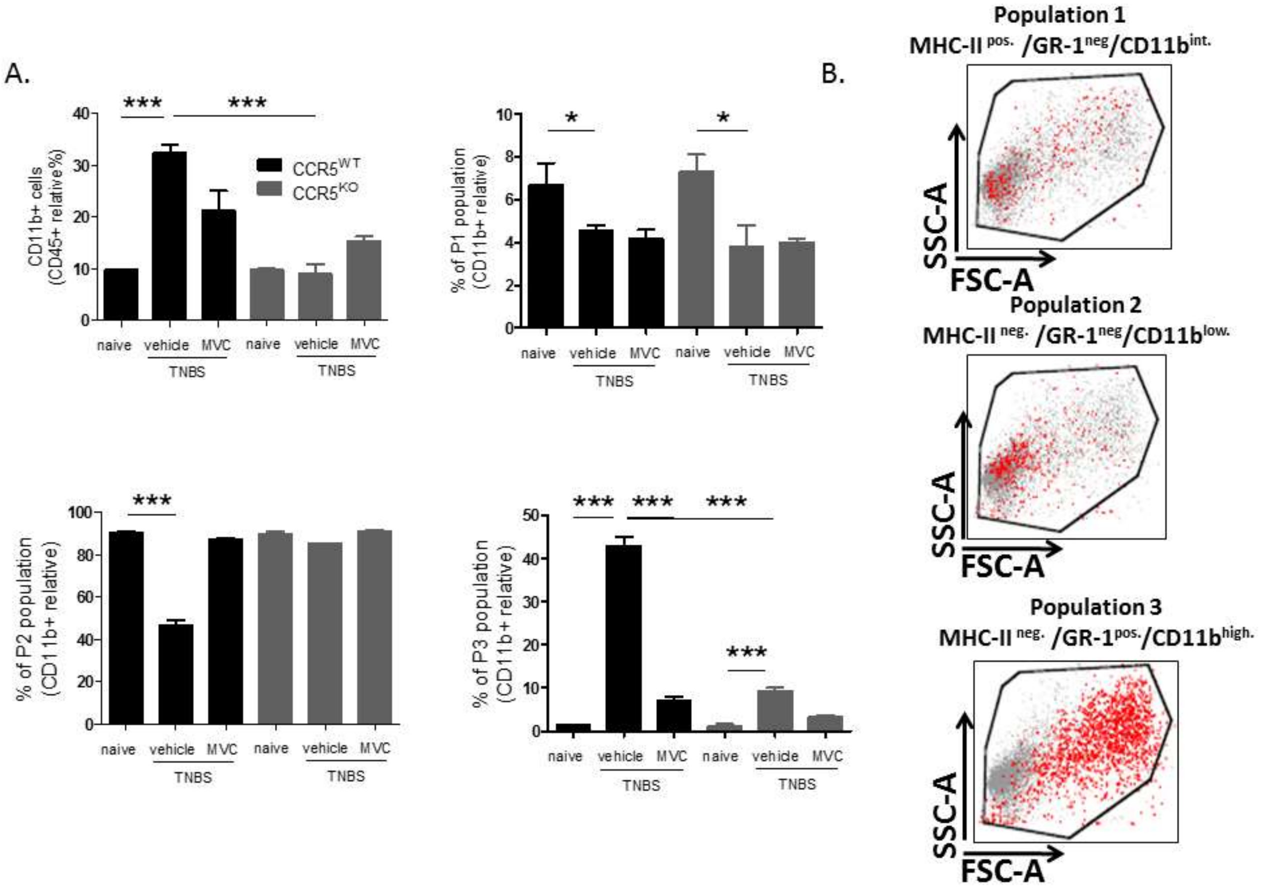

A.
***
***
CD11b+ cells
(CD45+ relative%)
CCR5WT
CCR5KO
naive vehicle MVC naive vehicle MVC
TNBS
TNBS
*
*
% of P1 population
(CD11b+ relative)
% of P2 population
(CD11b+ relative)
% of P3 population
(CD11b+ relative)
B.
Population 1
MHC-II pos. /GR-1neg/CD11bint.
Population 2
MHC-II neg. /GR-1neg/CD11blow.
Population 3
MHC-II neg. /GR-1pos./CD11bhigh.
SSC-A
FSC-A

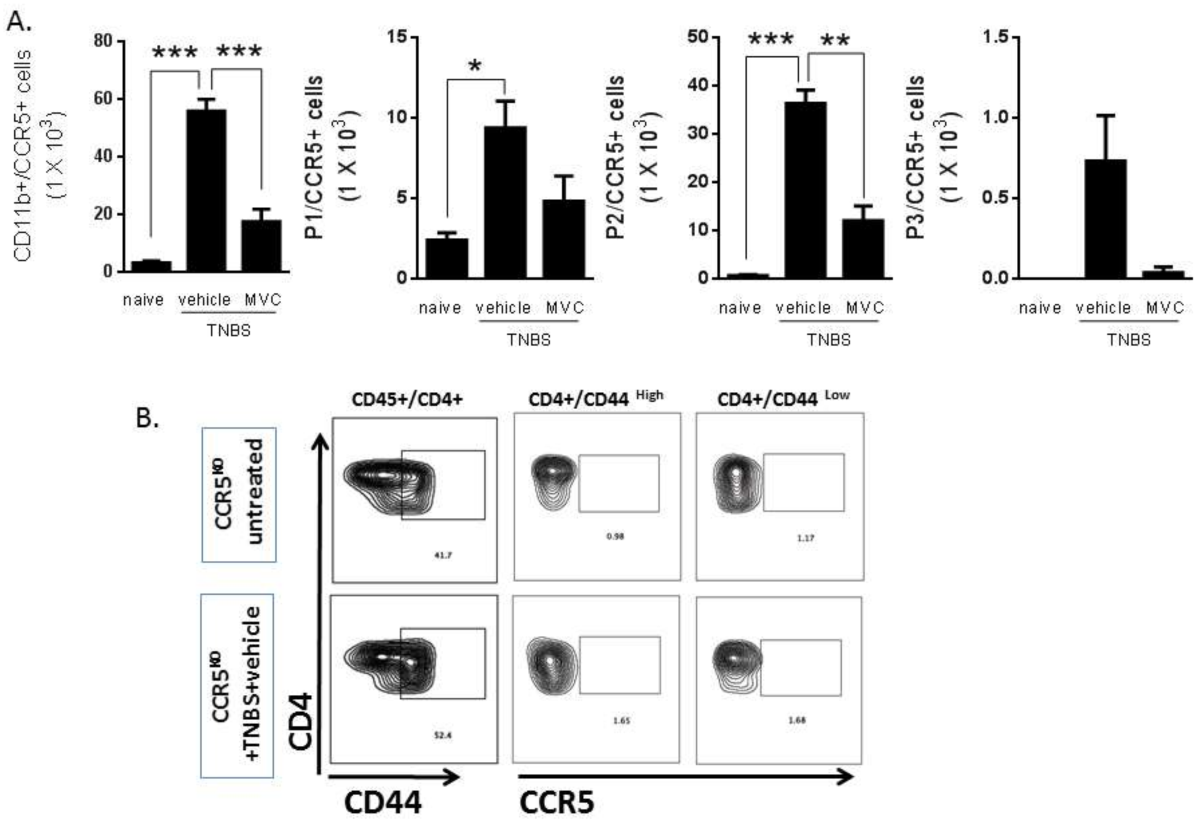

A.
CD11b+/CCR5+ cells (1 X 10^3)
P1/CCR5+ cells (1 X 10^3)
P2/CCR5+ cells (1 X 10^3)
P3/CCR5+ cells (1 X 10^3)
***
**
*
naive
vehicle
MVC
TNBS
B.
CD45+/CD4+
CD4+/CD44 High
CD4+/CD44 Low
CCR5KO untreated
CCR5KO +TNBS+vehicle
CD4
CD44
CCR5

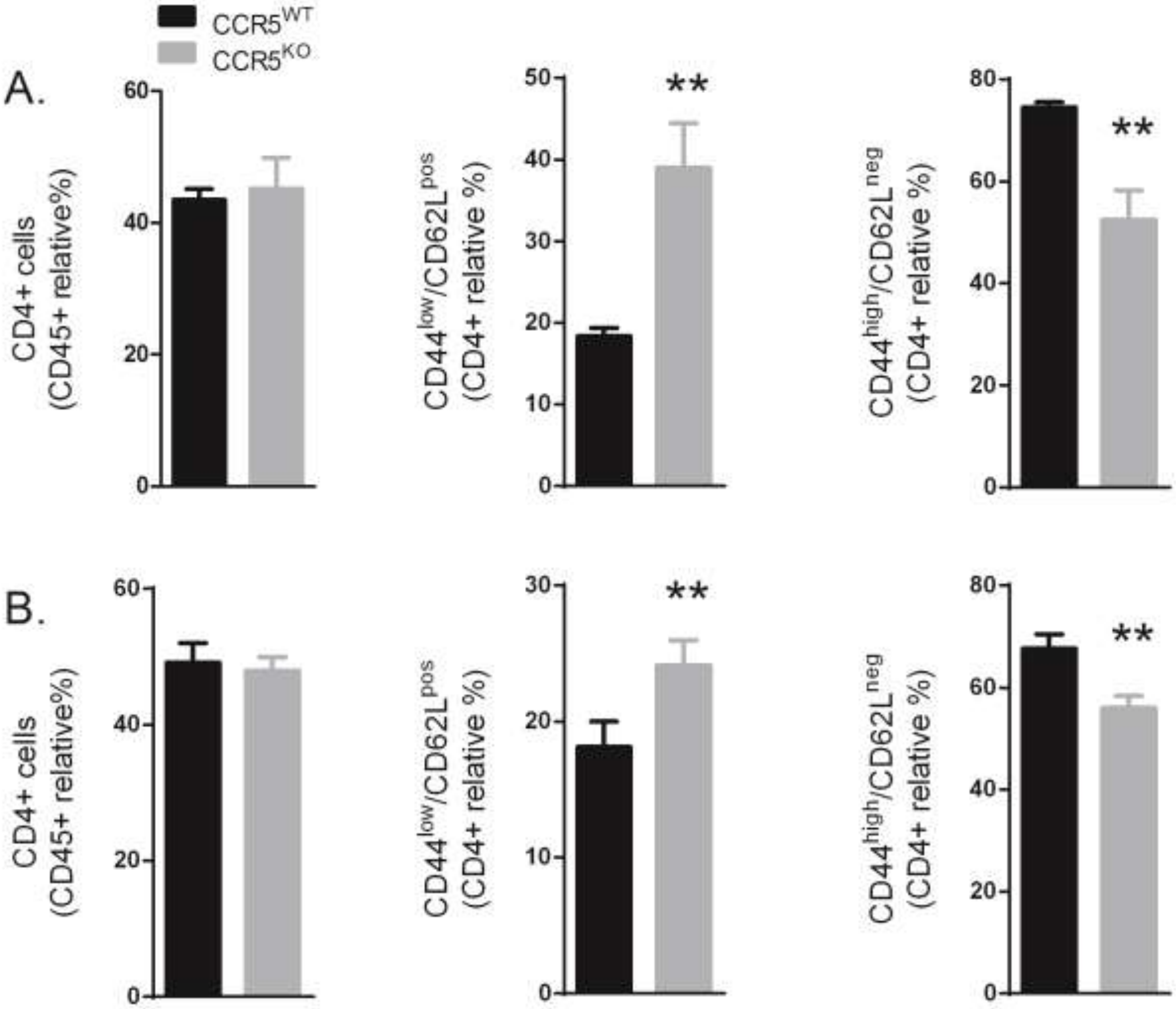

CCR5WT
CCR5KO
A.
CD4+ cells (CD45+ relative%)
60
40
20
0
CD44low/CD62Lpos (CD4+ relative %)
50
40
30
20
10
0
**
CD44high/CD62Lneg (CD4+ relative %)
80
60
40
20
0
**
B.
CD4+ cells (CD45+ relative%)
60
40
20
0
CD44low/CD62Lpos (CD4+ relative %)
30
20
10
0
**
CD44high/CD62Lneg (CD4+ relative %)
80
60
40
20
0
**

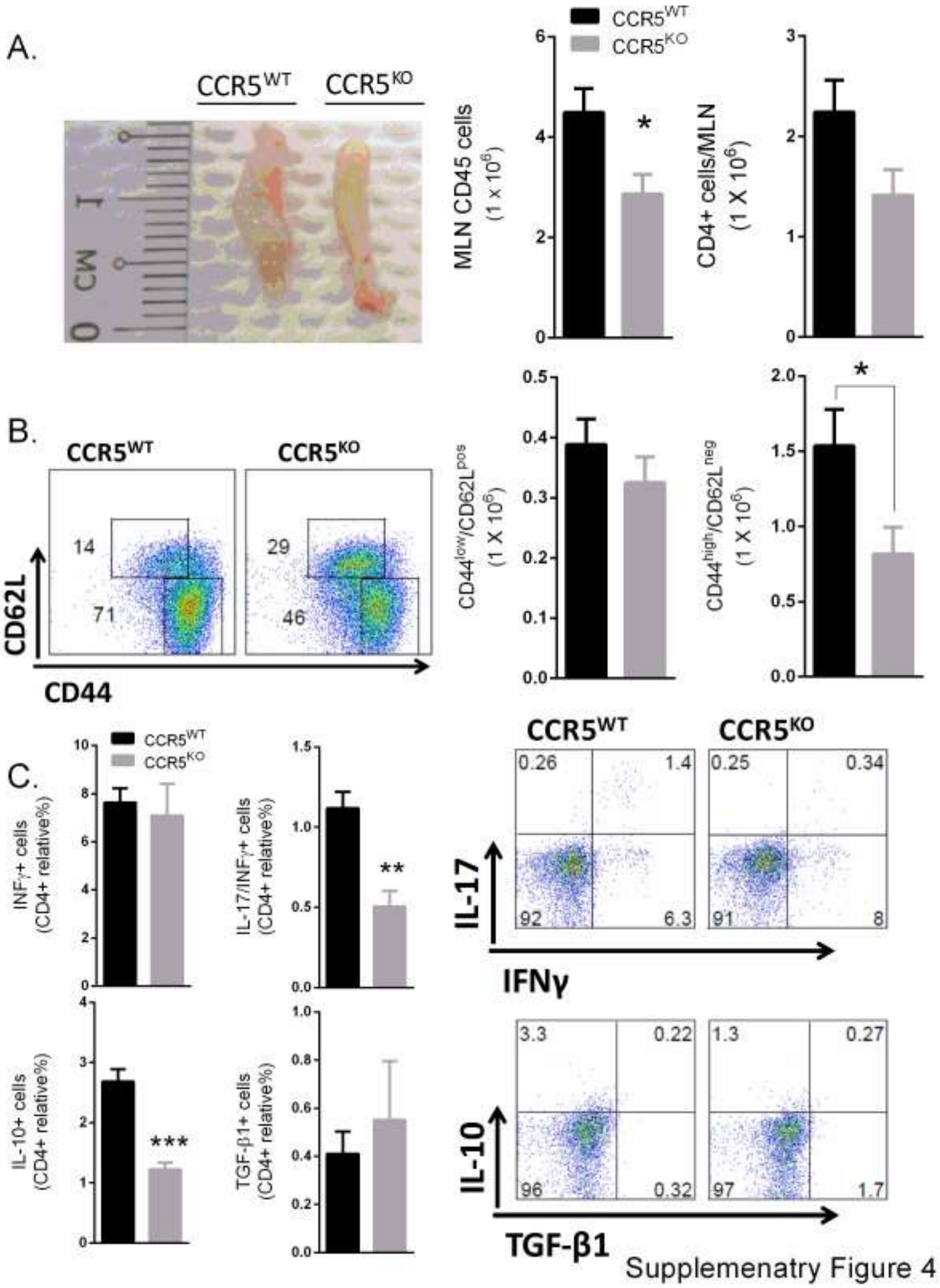

Supplemenatry Figure 4

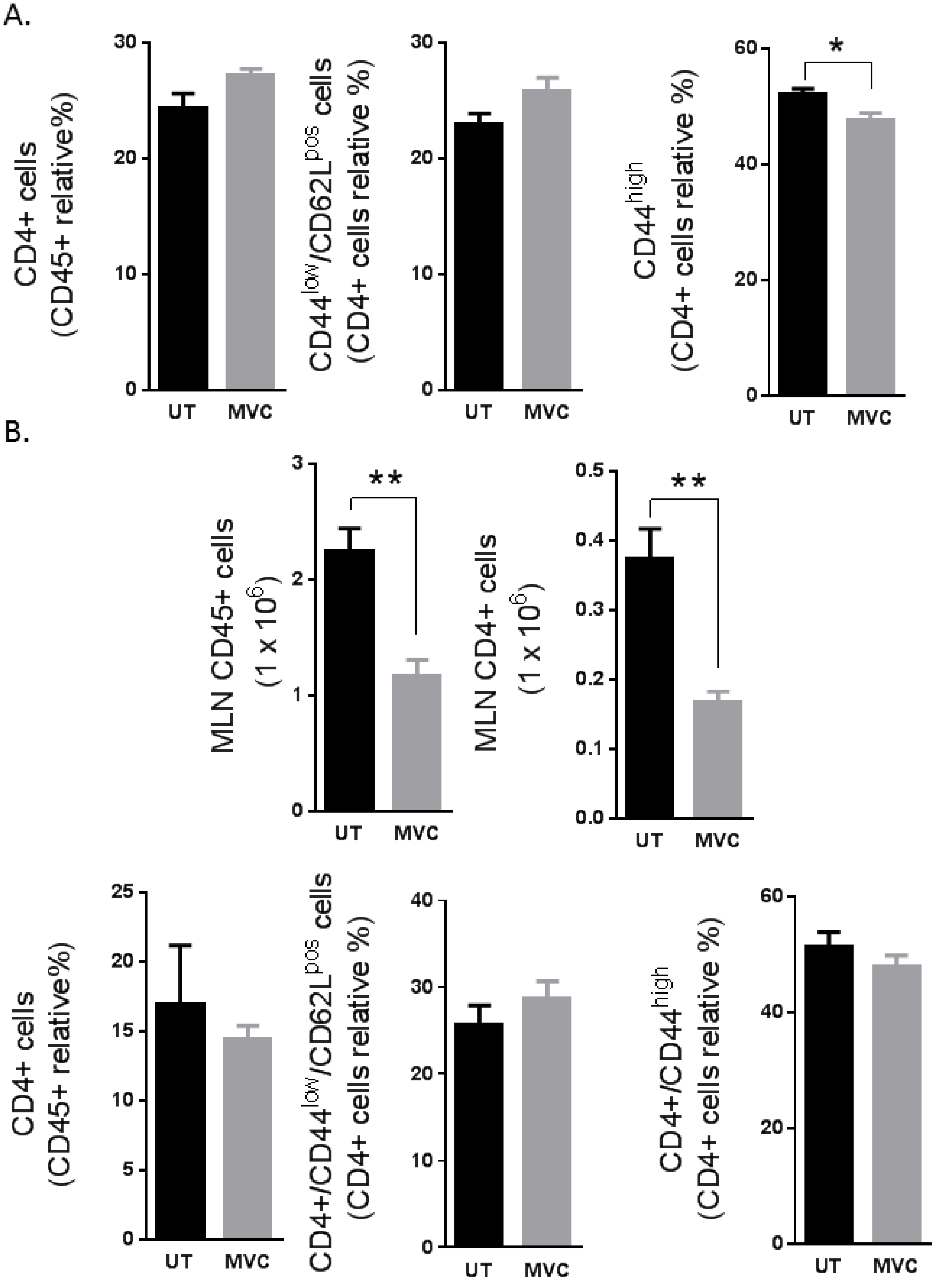

A.
CD4+ cells (CD45+ relative%)
30
20
10
0
UT
MVC
CD44low/CD62Lpos cells (CD4+ cells relative %)
30
20
10
0
UT
MVC
CD44high (CD4+ cells relative %)
*
60
40
20
0
UT
MVC
B.
MLN CD45+ cells (1 x 10^6)
**
3
2
1
0
UT
MVC
MLN CD4+ cells (1 x 10^6)
**
0.5
0.4
0.3
0.2
0.1
0.0
UT
MVC
CD4+ cells (CD45+ relative%)
25
20
15
10
5
0
UT
MVC
CD4+/CD44low/CD62Lpos cells (CD4+ cells relative %)
40
30
20
10
0
UT
MVC
CD4+/CD44high (CD4+ cells relative %)
60
40
20
0
UT
MVC
